# Primary biocompatibility tests of poly(lactide-co-glycolide)-(poly-L-orithine/fucoidan) core–shell nanocarriers

**DOI:** 10.1098/rsos.180320

**Published:** 2018-07-18

**Authors:** Duanhua Cai, Jingqian Fan, Shibin Wang, Ruimin Long, Xia Zhou, Yuangang Liu

**Affiliations:** 1 College of Chemical Engineering, Huaqiao University, Xiamen 361021, People's Republic of China; 2 Institute of Pharmaceutical Engineering, Huaqiao University, Xiamen 361021, People's Republic of China; 3 Fujian Provincial Key Laboratory of Biochemical Technology, Huaqiao University, Xiamen 361021, People's Republic of China

**Keywords:** layer-by-layer self-assembly, poly-ornithine, fucoidan, PLGA, anti-tumour, biocompatibility

## Abstract

Layer-by-layer (LbL) self-assembly is the technology used in intermolecular static electricity, hydrogen bonds, covalent bonds and other polymer interactions during film assembling. This technology has been widely studied in the drug carrier field. Given their use in drug delivery systems, the biocompatibility of these potential compounds should be addressed. In this work, the primary biocompatibility of poly(lactide-co-glycolide)-(poly-L-orithine/fucoidan) [PLGA-(PLO/fucoidan)] core–shell nanoparticles (NPs) was investigated. Atomic force microscopy revealed the PLGA-(PLO/Fucoidan)_4_ NPs to be spherical, with a uniform size distribution and a smooth surface, and the NPs were stable in physiological saline. The residual amount of methylene chloride was further determined by headspace gas chromatography, in which the organic solvent can be volatilized during preparation. Furthermore, cell viability, acridine orange/ethidium bromide staining, haemolysis and mouse systemic toxicity were all assessed to show that PLGA-(PLO/fucoidan)_4_ NPs were biocompatible with cells and mice. Therefore, these NPs are expected to have potential applications in future drug delivery systems.

## 1. Introduction

Nanocarriers, which have specific properties such as nano-size and controlled drug delivery behaviour, have been widely studied in drug delivery systems. Compared with other types of nanocarriers, layer-by-layer (LbL) self-assembled carriers have a better controlled release effect mainly due to the LbL structure.

The LbL self-assembly technique can combine different polyelectrolyte materials and nanoparticles (NPs) to construct a carrier with an ultra-thin film multilayer structure. Through the precise control of various parameters, such as membrane structure, film thickness, and chemical and mechanical properties, it can deliver and release the drug into tumour cells [1–3]. The controlled release effect of LbL self-assembled drug carriers is primarily manifested through the following: (1) the environment-dependent multilayer assembly characteristics determine the controlled release of the drug by regulating the lamellar membrane structure; (2) the assembly characteristics of a variety of polymers include the inclusion of hydrolysis, enzymolysis, stimulus response, etc. to regulate drug release; and (3) the preparation method of LbL self-assembly technology by designing the spatial distribution of drugs allows multilayer films to achieve controllable drug release [4,5].

Among the various polyelectrolyte membrane materials, the newly developed materials poly-l-ornithine (PLO) and fucoidan have been reported by our group recently. PLO is a low-immunogenic polycationic compound, which has a variety of biological activities and good biosecurity [6–9], and can improve the mechanical properties of particle strength and reduce particle collapse during transport. Fucoidan is a polysaccharide containing a variety of biological activities, including anticoagulant, hypolipidaemic, anti-tumour and anti-virus properties, as well as the ability to enhance immune function. In terms of anti-tumour activity, fucoidan can induce tumour cell apoptosis [10] and has been shown to affect the formation of tumour cells in colon cancer [11]. Many studies have reported that fucoidan has anti-tumour activity against various human cancers [12]. For example, it can be used as a potential anti-cancer drug in both colon and breast cancer [13]. Thus, the composition of these two polyelectrolyte materials provides new possibilities for the construction of layers of self-assembled drug carriers.

As a potential new drug carrier, poly(lactide-co-glycolide) [PLGA]-(PLO/fucoidan) core–shell nanocarriers should not only possess drug-loading and release properties [14], but also good biocompatibility. Herein, the surface morphology and residual organic solvents were assessed. Cytotoxicity was analysed with the cell counting kit-8 (CCK-8) assay and acridine orange/ethidium bromide (AO/EB) staining, and a haemolysis test. Finally, overall systemic toxicity was also assessed in the mouse. We anticipate that this new drug carrier will have a bright future in the drug delivery field.

## 2. Material and methods

### 2.1. Materials

LbL self-assembled NPs were prepared as previously described [14]. Briefly, the PLGA NPs were prepared by the O/W emulsion–solvent evaporation method, and then PLO and fucoidan were deposited layer by layer alternately on the surface of the substrate core by electrostatic interaction. The CCK-8 kit BS350B was purchased from Biosharp Biotech (China). AO and EB solutions were kindly supplied by Beijing Solarbio. The human breast cancer cell line MCF-10A was provided by the Chinese Academy of Sciences Shanghai Cell Bank. SPF grade mice were received from Shanghai Slack Animal Experimental Center. Other chemicals were received from Sinopharm Chemical Reagent Ltd (China).

### 2.2. Observation of nanomorphology by atomic force microscopy

Freeze-dried PLGA-(PLO/fucoidan)_4_ NPs were dispersed in ethanol and the droplets were fixed to the mica sheet. The Bruker probe was installed and the tapping mode selected to observe the nanomorphology with atomic force microscopy (AFM; SCANASYST, Bruker Company, America).

### 2.3. Stability test

PLGA-(PLO/fucoidan)_4_ NPs were dispersed in physiological saline at 37°C. To investigate the stability of the NPs in physiological saline, changes in the diameter of the NPs were detected with an NP size potentiometer (Zetasizer nano, Malvern Instruments, Worcestershire, UK) after the respective time intervals (0, 24, 48 and 72 h).

### 2.4. Headspace gas chromatography detection of residual organic solvents

A simple and sensitive gas chromatographic method has been developed and validated for detection of residual organic solvents [15]. The conditions for detecting the residual organic solvents were set as follows: chromatographic column type, DB-624 capillary column; sample inlet and detector temperatures, 200°C and 250°C, respectively. The column temperature was programmed with an initial temperature of 40°C, initial time of 7 min, program rate of 10°C min^−1^, final temperature of 250°C and final time of 2 min. Then, the dichloromethane (DCM) reference was diluted with dimethylformamide (DMF) to make a reference solution of 60 µg ml^−1^. Finally, 0.1 g accurately weighed PLGA NPs sample was added to the headspace bottle, 2 ml DMF was added and placed in a headspace sampler, followed by 30 min at 80°C and headspace injection of a divided-flow type with a flow rate of 1.0 ml min^−1^ and split ratio of 20 : 1. Residual organic solvent was detected by headspace gas chromatography (HS-GC; HP-6890N, Agilent Corporation, America).

### 2.5. Cell counting kit-8 assay

The cell counting kit-8 (CCK-8) method was used to determine the cytotoxicity [16]. Breast epithelium cells (MCF-10A cell line was kindly supplied by the Institute of Cell Resource Center, Chinese Academy of Sciences, Shanghai) were cultured in Dulbecco's modified Eagle medium containing 10% fetal bovine serum. Cells were inoculated at a density of 5 × 10^5^ cells per well in 96-well plates and incubated for 48 h at 37°C and 5% CO_2_ to evaluate the effects of PLGA and PLGA-(PLO/fucoidan)_4_ NPs on the cytotoxicity of MCF-10A cells. The cells were cultured for 24 h, the medium was aspirated and the following was added: fresh medium (negative control), PLGA or LbL NPs (5, 10, 25, 50 and 100 µg ml^−1^), or 0.64% phenol at 100 µl well^−1^. After 72 h, the CCK-8 assay was used to evaluate dose-dependent toxicity according to manufacturer's instructions. In addition, time-dependent toxicity studies of cells with fresh medium (negative control and blank), PLGA or LbL NPs (nano concentration: 100 µg ml^−1^), or 0.64% phenol added at 100 µl well^−1^ were performed at 24, 48 and 72 h. The relative growth rate of cell was calculated according to the following equation to evaluate the cytotoxicity of the material.

2.1 $$\text{RGR}(\%)=\frac{\text{Test groupO}{\text{D}}_{450}-\text{Blank groupO}{\text{D}}_{450}}{\text{Negative groupO}{\text{D}}_{450}-\text{Blank groupO}{\text{D}}_{450}}.$$

### 2.6. Acridine orange/ethidium bromide staining

Cells in logarithmic growth were seeded in 24-well plates at a concentration of 5 × 10^4^ cells ml^−1^. After 24 h of culture, 1 ml of PLGA and PLGA-(PLO/fucoidan)_4_ NPs were added to each well at 100 µg ml^−1^, and co-cultured for 72 h. The cells were digested with EDTA and stained with AO/EB. Cell morphology was observed with a confocal laser scanning microscope (LSM710, Zeiss, Germany).

### 2.7. Haemolysis test

Fresh rabbit blood from female rabbits (2 kg; provided by the Shanghai Slake Animal Laboratory Center) was collected by intravenous injection to prepare fresh anticoagulated rabbit whole blood. The amount was adjusted with saline to prepare fresh anticoagulant-saline stock solution with an absorbance at 545 nm of 0.8 ± 0.3. Different concentrations of PLGA and PLGA-(PLO/fucoidan)_4_ NP suspensions (25, 50 and 100 µg ml^−1^) were prepared with 0.9% saline. Saline was used as a negative control, distilled water as a positive control and 0.2 ml diluted anticoagulant rabbit blood serum was added to each of the above groups. The samples were mixed evenly and incubated in a water bath at 37°C for 60 min, centrifuged at 2500 r.p.m. for 5 min and the absorbance of the supernatant was measured at 545 nm. The rate of haemolysis of the cells, expressed as a percentage (%), was calculated according to equation (2.2), and the blood compatibility of the materials was evaluated according to the standard requirements [17–19].

2.2 $$\text{Haemolysis rate }(\%)=\frac{\text{Sample to be testedO}{\text{D}}_{\mathrm{mean}}-\text{Negative groupO}{\text{D}}_{\mathrm{mean}}}{\text{Positive groupO}{\text{D}}_{\mathrm{mean}}-\text{Negative groupO}{\text{D}}_{\mathrm{mean}}}.$$

### 2.8. Acute systemic toxicity in mice

Nano-suspensions were prepared according to the requirements of the systemic toxicity test in the Biological Evaluation Criteria of Medical Devices (GB/T 16886. 12-2005/ISO 10993-12: 2002) and 24 SPF mice (half of female and male) weighing 17–20 g were on average divided by gender into four groups. The animals were injected intraperitoneally with PLGA NP suspension (30 ml kg^−1^), LbL NP nano-suspension (30 ml kg^−1^), normal saline or 6.4% phenol solution. To investigate the changes in overall weight, the mice were weighed at 24, 48 and 72 h after injection, and the general status and toxicity were observed. According to the requirements (GB/T 16886. 11-2011/ISO 10933-11: 2006), material toxicity is classified as non-toxic, mildly toxic, moderately toxic, severely toxic and death, according to the degree of the symptoms of poisoning. Haematoxylin and eosin (HE) staining was performed to observe the nuclei and cytoplasm. The preparation of mouse liver tissue HE-stained sections was performed as described previously [20–22].

## 3. Results and discussion

### 3.1. Observation of nanomorphology by atomic force microscopy

**Figure 1.**
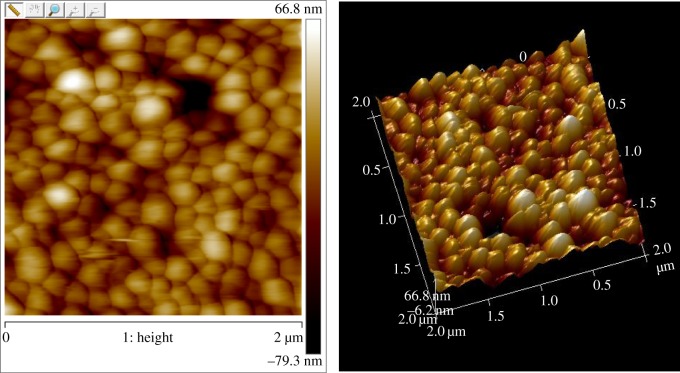
Atomic force microscopy images of poly(lactide-co-glycolide) (PLGA)-(poly-L-orithine [PLO]/fucoidan)_4_ nanoparticles (NPs).

AFM can be used to observe the surface morphology, surface adhesion, friction, elasticity and plasticity of samples. The AFM results revealed that the LbL NPs are spherical, with a uniform distribution of particle size and adhesion between the particles (figure 1). From the height map, it can be seen that the particle surface exhibits a certain degree of roughness after being wrapped by the polyelectrolyte. This structure is conducive to the encapsulation of drugs with larger surface area.

### 3.2. Nanoparticle stability

**Figure 2.**
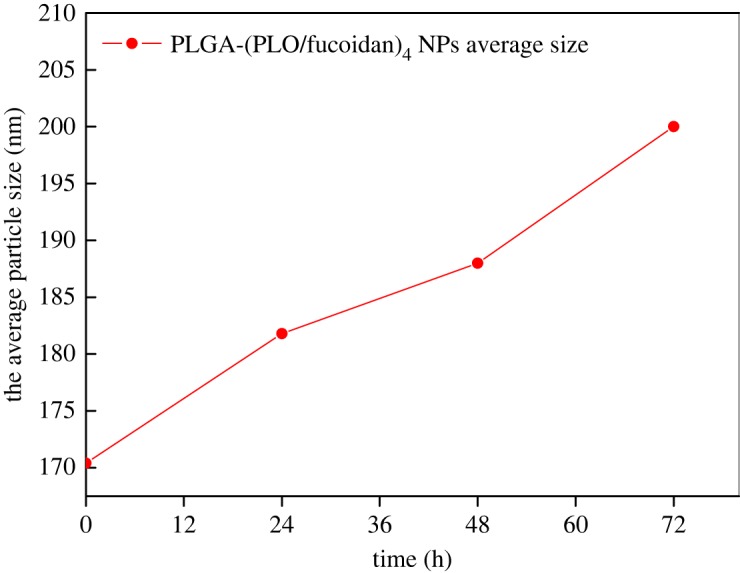
The mean diameter of PLGA-(PLO/fucoidan)_4_ NPs after incubation in saline (37°C) at 0, 24, 48 and 72 h.

Figure 2 shows the change in size of PLGA-(PLO/fucoidan)_4_ NPs incubated in saline at 37°C for 3 days. The particle size of self-assembled NPs increased 11.40 nm on day 1, 6.20 nm on day 2 and 12.00 nm on day 3, for a total of 29.60 nm, which accounts for 17.37% of the initial particle size. The results indicated that the particles exhibited good mechanical properties in saline, with a low swelling rate and no evidence of particle breakage, which showed the stability and potential application in the drug delivery system.

### 3.3. Headspace gas chromatography detection of residual organic solvents

**Figure 3.**
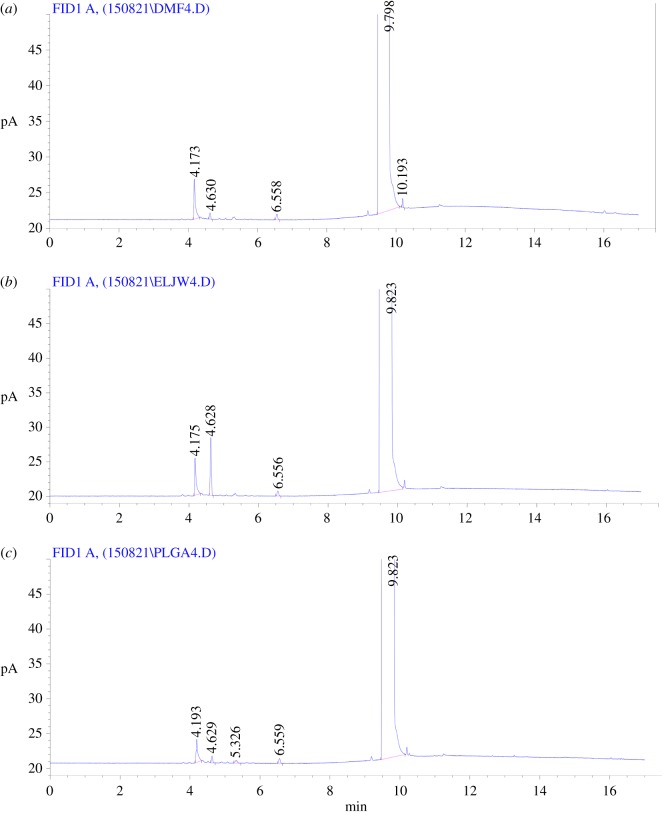
The content of dichloromethane (DCM) in PLGA NPs by headspace gas chromatography. (*a*) Dimethylformamide; (*b*) DCM and (*c*) PLGA NPs.

PLGA NPs were prepared by ultrasonic emulsification, and DCM was chosen as the oil phase for emulsification. Therefore, the residual amount of DCM contained in the material was determined by HS-GC. We found that the retention time of 5.4 min was recorded for DCM. Furthermore, DCM residue was less than the minimum detection limit (0.06%) of the instrument, which did not detect residual methylene chloride, which is much lower than that of the USP 467 pharmacopoeia prescribed 600 ppm. This indicates the 3 h of stirring is sufficient to fully volatilize the organic solvent (figure 3).

### 3.4. Analysis of cell proliferation with the cell counting kit-8 assay

#### 3.4.1. Cell morphology

**Figure 4.**
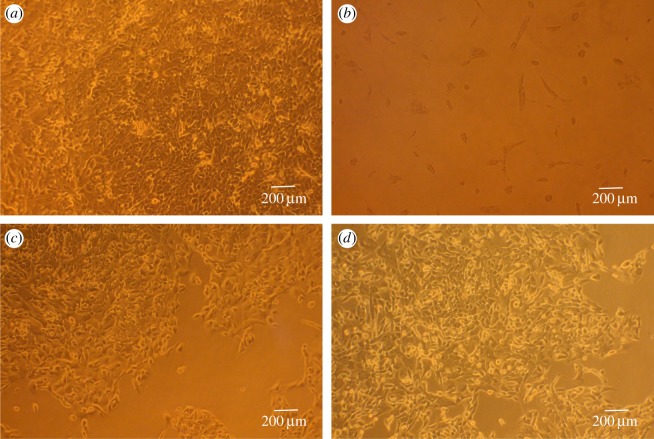
Optical micrograph of MCF-10A cells cultured for 72 h with 100 µg ml^−1^ PLGA or LbL NPs. (*a*) Fresh medium (control), (*b*) positive control, (*c*) PLGA NPs and (*d*) LbL NPs.

Figure 4 shows a microscopic image of PLGA and LbL NPs at 100 µg ml^−1^ co-cultured with MCF-10A cells for 72 h. Cells in fresh medium (negative control) grew well as filamentous or polygonal cells with large and round nuclei, abundant cytoplasm and clear nuclei (figure 4*a*), while cell density was decreased, nuclei became blurred, and cells were solid or round and in the 0.64% phenol group (figure 4*b*). The cells containing PLGA (figure 4*c*) and LbL NPs (figure 4*d*) grew well, with normal morphology.

#### 3.4.2. Cell proliferation

**Figure 5.**
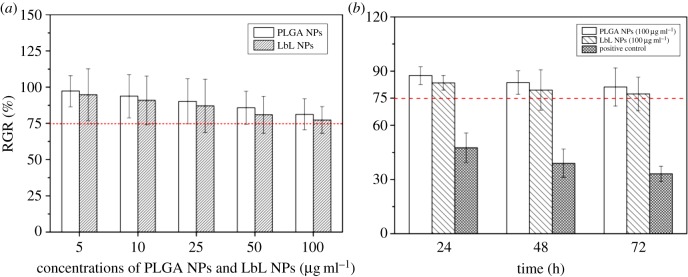
RGR of MCF-10A cells cultured in PLGA and LbL NP suspensions. (*a*) Different concentrations (5, 10, 25, 50 and 100 µg ml^−1^) and (*b*) different times (24, 48 and 72 h).

Figure 5*a* shows the rate of proliferation of PLGA and LbL NPs co-cultured with MCF-10A cells for 72 h at different concentrations (5, 10, 25, 50 and 100 µg ml^−1^). With increasing NP concentration, the rate of proliferation was reduced. When the concentration was 100 µg ml^−1^, cell proliferation was 81.24% and 77.39% in the PLGA and LbL NP groups, respectively. Figure 5*b* shows the proliferation rates of PLGA and LbL NPs at 100 µg ml^−1^ in MCF-10A cells for 24, 48 and 72 h. Proliferation was inhibited with time: the proliferation of MCF-10A cells incubated with PLGA NPs was reduced from 87.52% to 81.24%, while the MCF-10A cells incubated with LbL NPs decreased from 83.50% to 77.40%. At the same time, the addition of LbL NPs inhibited proliferation more efficiently than the addition of PLGA NPs, which is due to the self-assembled nano-encapsulated polyelectrolyte materials, which may have an impact on cell growth. However, the difference between the two types of NPs is small and the rate of proliferation was greater than 75% and the toxicity evaluation was level 1 in both conditions, indicating that the materials can be considered biologically safe. The cytotoxicity of PLO was weakened by PEGylation due to the conjugated PEG chains shielding the positively charged amine groups of it [23]; therefore, the fabrication of LbL NPs with PEGylated PLO should be considered, which would be promising for the development of anti-cancer drug delivery systems.

### 3.5. Acridine orange/ethidium bromide staining to observe apoptosis

**Figure 6.**
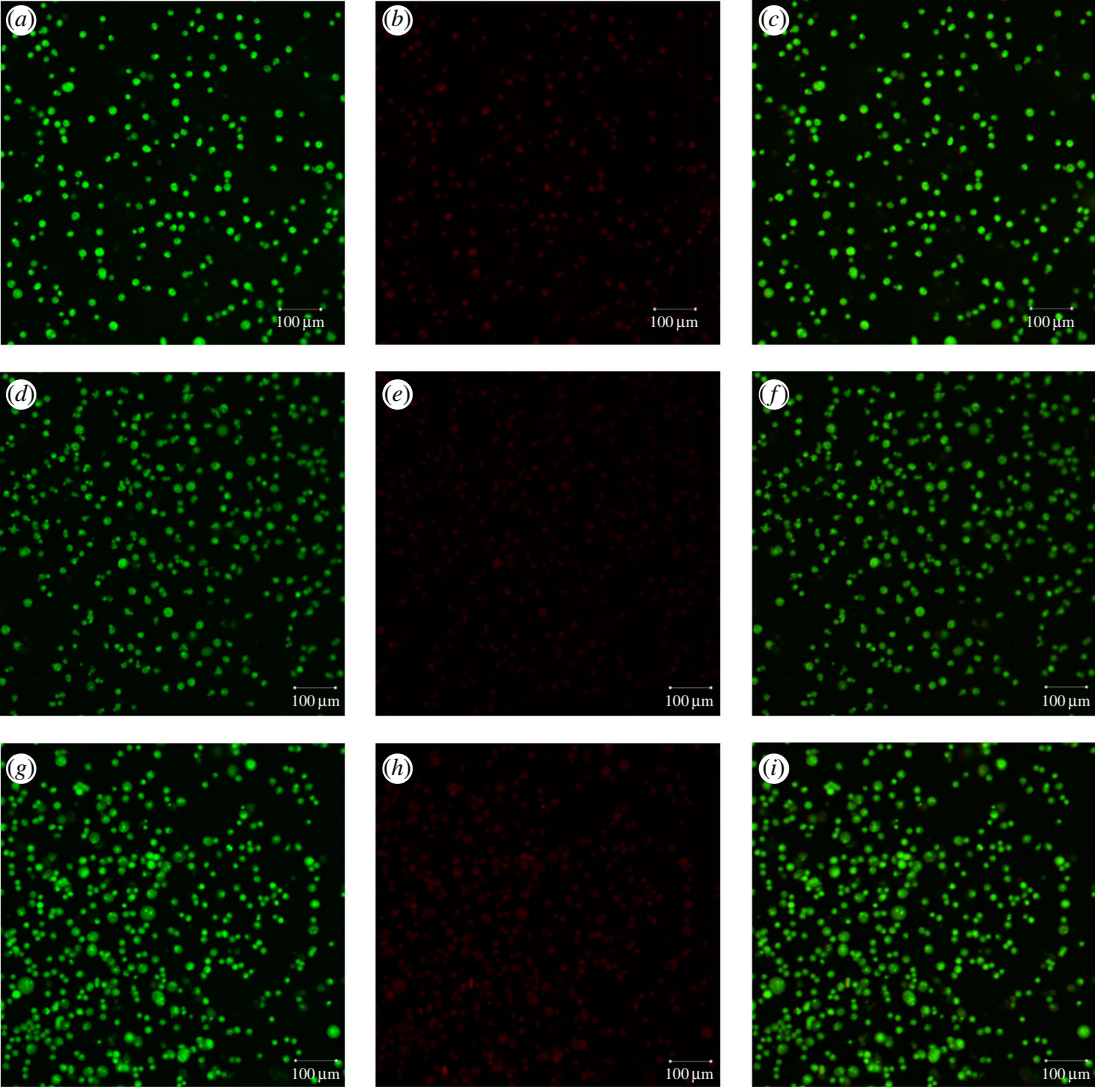
Acridine orange/ethidium bromide (AO/EB) dual staining of MCF-10A cells. (*a*–*c*) fresh medium (control); (*d*–*f*) PLGA NPs and (*g*–*i*) LbL NPs.

Figure 6 shows the confocal laser scanning image of MCF-10A cells stained with AO/EB. The cells shown are in a normal growth state, round with green fluorescence, with no obvious apoptosis. This suggests that both PLGA and LbL NPs are biologically safe and have no significant effect on the morphology of MCF-10A cells.

### 3.6. Haemolysis test

**Figure 7.**
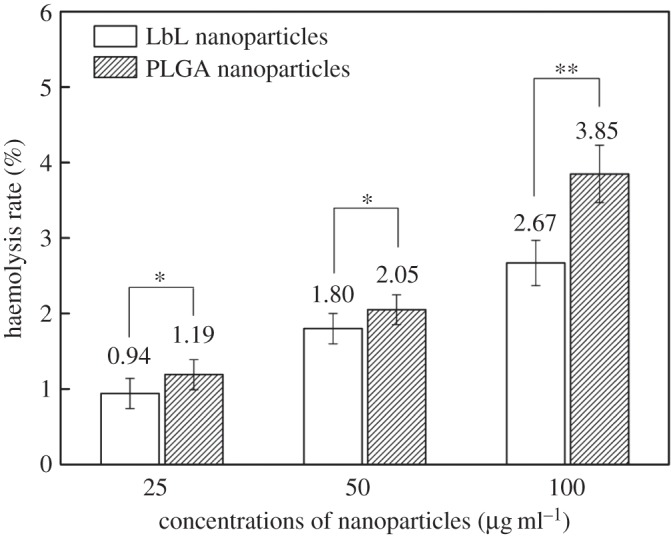
Haemolysis test.

Haemolysis refers to the phenomenon of red blood cell rupture and dissolution. A haemolysis test is used to evaluate the blood compatibility of materials. Figure 7 shows the effect of different concentrations of PLGA and LbL NPs (25, 50 and 100 µg ml^−1^) on fresh rabbit blood. With an increase in material concentration, the rate of haemolysis also increased, with the PLGA NP group significantly higher than the LbL NP group. Haemolysis test results illustrate that the polyelectrolyte material is wrapped to help improve the biocompatibility of PLGA.

### 3.7. Acute systemic toxicity in mice

The acute toxicity test is performed to observe experimental animals after injection for symptoms of toxicity and to determine the extent of the symptoms over time. Intraperitoneal injection of experimental mice was performed according to the standard (GB/T 16886. 11-2011/ISO 10933-11: 2006). The samples are absorbed through the portal vein and circulated through the liver before reaching the systemic circulation. Therefore, HE staining of the liver and kidney of the experimental mice was performed to observe toxicity.

**Figure 8.**
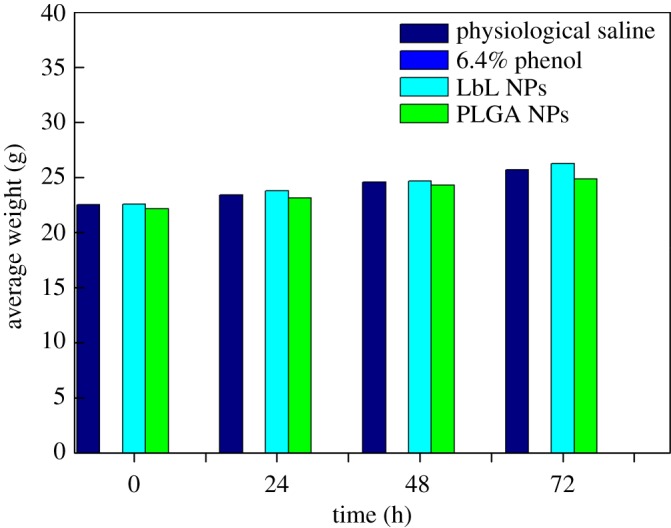
Weight gain in mice.

**Figure 9.**
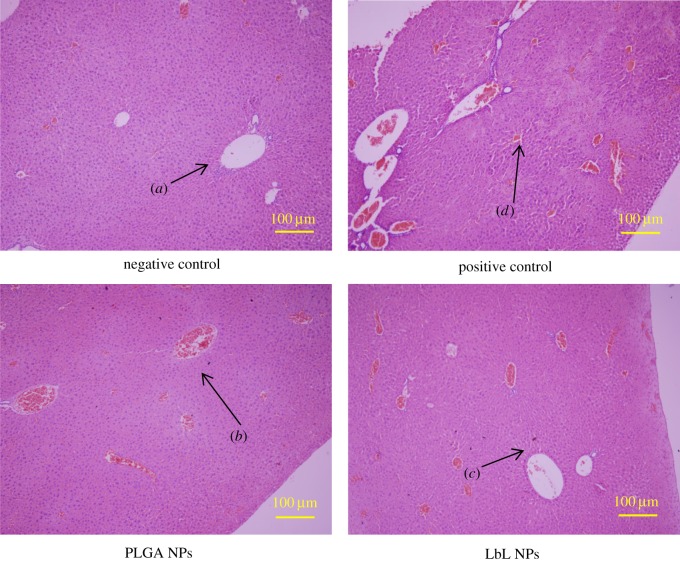
Haematoxylin and eosin-stained section of mouse liver tissue after injection.

The whole-body acute toxicity test showed that the growth condition of the mice in the material group was quite normal and no obvious toxicity was observed. These results were consistent with the results of the group of mice administered saline, which exhibited normal consumption of food and water and regular activities. No death was observed during the observation period of 72 h. Mice injected with 6.4% phenol had significant toxic manifestations, including shortness of breath, difficulty breathing, whole-body convulsions, tremulous bursts, exophthalmos and then gradually turning purple, which turned into a jerky reflex with rough hair until death. Figure 8 shows the changes in body weight of the animals. The positive control mice died after injection, demonstrating that 6.4% phenol had obvious toxic effects on mice. The overall weight of the negative control, PLGA NP (10 mg ml^−1^) and the LbL NP (10 mg ml^−1^) groups of mice increased over time, which could indicate that the health of the mice was sound and that the material did not cause toxic effects. Therefore, our results suggest that PLGA and LbL NPs exhibit good biosafety. Analysis of animal liver pathology is shown in figure 9. The liver is the body's largest digestive gland, which secretes bile and is important for metabolism and detoxification in the body. The liver consists of hepatic lobules (hepatocytes, bile ducts and the hepatic sinusoid), the portal area and hepatic vessels (hepatic portal vein and hepatic artery). The lobuli hepatis of the group administered PLGA and LbL NPs and the negative group showed a polygon with a central vein in the axis of the hepatic lobule (figure 9*a*), and hepatocytes (figure 9*c*) radiated around the central vein to the periphery to form hepatocyte plates. The irregular interstices between the hepatocyte plates are hepatic sinusoids (figure 9*b*), which are connected to the network in the hepatic lobules, where the blood flows into the central vein. Nevertheless, cells of the group of mice administered 6.4% phenol exhibited deformation; the liver cell gap became larger, and was accompanied by the occurrence of punctate necrosis. By comprehensive comparison, we could summarize that PLGA and LbL NPs exhibited no damage to the liver of mice and the materials exhibited good biocompatibility.

## 4. Conclusion

Patients with advanced cancer who received fucoidans via oral administration exhibited a significant reduction in major pro-inflammatory cytokines after two weeks [24]. This is the first research study to provide evidence of the anti-inflammatory effects of fucoidans on patients with advanced cancer. Fucoidan may form polyelectrolyte complexes with chitosan in the preparation of NPs for the delivery of growth factors, drugs and naturally occurring bioactive compounds [25,26]. However, chitosan/fucoidan NP complexes become unstable under normal physiological conditions. NPs prepared with the LbL self-assembly technique, PLO and fucoidan, are assembled via electrostatic interactions, not only retaining their biological activity but also increasing the stability of the carrier. Experiments show that the PLGA-(PLO/fucoidan)_4_ NPs have better biocompatibility, thus it appears that the LbL self-assembly technology will offer a promising approach for the development of biocompatibility drugs in the future. It will be necessary to perform pharmacokinetic studies of the *in vivo* anti-cancer activity of drug-loaded PLGA-(PLO/fucoidan)_4_ NPs to obtain reliable evidence for the application of PLGA-(PLO/fucoidan)_4_ NPs in cancer treatment.

Now, LbL self-assembly technology has a lot of application prospects in cancer treatment, especially in the optimization of such a system. For example, with material modification, co-delivery of multiple chemotherapeutic drugs or genes, as well as in conjunction with target ligands, the LbL nanocarrier can be significantly optimized. Better biocompatibility and anti-tumour efficacy will be improved with optional matching of materials and structure adjustment.

## Data accessibility

All data are included within the article.

## References

[1] Das BP, Tsianou M 2016 From polyelectrolyte complexes to polyelectrolyte multilayers: electrostatic assembly, nanostructure, dynamics, and functional properties. Adv. Colloid Interface Sci. 244, 71–89. (10.1016/j.cis.2016.12.004) 28499602

[2] Freag MS, Elnaggar YS, Abdelmonsif DA, Abdallah OY 2016 Layer-by-layer-coated lyotropic liquid crystalline nanoparticles for active tumor targeting of rapamycin. Nanomedicine (Lond) 11, 2975–2996. (10.2217/nnm-2016-0236) 27785978

[3] Bozdoğan B, Akbal Ö, Çelik E, Türk M, Denkbaş EB 2017 Novel layer-by-layer self-assembled peptide nanocarriers for siRNA delivery. RSC Adv. 7, 47 592–47 601. (doi:10.1039/C7RA08460A)

[4] Xu C, Zhang C, Wang Y, Li L, Li L, Whittaker AK 2017 Controllable synthesis of a novel magnetic core-shell nanoparticle for dual-modal imaging and pH-responsive drug delivery. Nanotechnology 28, 495101 (10.1088/1361-6528/aa929b) 29019341

[5] Lynn DM 2007 Peeling back the layers: controlled erosion and triggered disassembly of multilayered polyelectrolyte thin films. Adv. Mater. 19, 4118–4130. (10.1002/adma.200701748)

[6] Balaji S, Zhou Y, Ganguly A, Opara EC, Soker S 2016 The combined effect of PDX1, epidermal growth factor and poly-L-ornithine on human amnion epithelial cells' differentiation. BMC Dev. Biol. 16, 8 (10.1186/s12861-016-0108-y) 27068127 PMC4828805

[7] Ge H et al. 2015 Poly-L-ornithine promotes preferred differentiation of neural stem/progenitor cells via ERK signalling pathway. Sci. Rep. 5, 15 535 (10.1038/srep15535) PMC4622086 26503112

[8] Wajsenzon IJ, Carvalho LA, Biancalana A, da Silva WAB, dos Santos Mermelstein C, de Araujo EG, Allodi S 2016 Culture of neural cells of the eyestalk of a mangrove crab is optimized on poly-L-ornithine substrate. Cytotechnology 68, 2193–2206. (10.1007/s10616-015-9942-1) 26779908 PMC5023563

[9] Liberio MS, Sadowski MC, Soekmadi C, Davis RA, Nelson CC 2014 Differential effects of tissue culture coating substrates on prostate cancer cell adherence, morphology and behavior. PLoS ONE 9, e112112 (10.1371/journal.pone.0112122.eCollection%202014) 25375165 PMC4223027

[10] Alekseyenko TV, Zhanayeva SY, Venediktova AA, Zvyagintseva TN, Kuznetsova TA, Besednova NN, Korolenko TA 2007 Anti-tumor and anti-metastatic activity of fucoidan, a sulfated polysaccharide isolated from the Okhotsk Sea *Fucus evanescens* brown alga. Bull. Exp. Biol. Med. 143, 730–732. (10.1007/s10517-007-0226-4) 18239813

[11] Fitton JH 2011 Therapies from fucoidan; multifunctional marine polymers. Mar. Drugs 9, 1731–1760. (10.3390/md9101731) 22072995 PMC3210604

[12] Wu L, Sun J, Su X, Yu Q, Yu Q, Zhang P 2016 A review about the development of fucoidan in antitumor activity: progress and challenges. Carbohydr. Polym. 154, 96–111. (10.1016/j.carbpol.2016.08.005) 27577901

[13] Atashrazm F, Lowenthal RM, Woods GM, Holloway A, Dickinson J 2015 Fucoidan and cancer: a multifunctional molecule with anti-tumor potential. Mar. Drugs 13, 2327–2346. (10.3390/md13042327) 25874926 PMC4413214

[14] Fan J, Liu Y, Wang S, Liu Y, Li S, Long R, Zhang R, Kankala RK 2017 Synthesis and characterization of innovative poly(lactide-co-glycolide)-(poly-L-ornithine/fucoidan) core–shell nanocarriers by layer-by-layer self-assembly. RSC Adv. 7, 32 786–32 794. (10.1039/C7RA04908K)

[15] Siddiqui MR, Singh R, Bhatnagar A, Kumar J, Chaudhary M 2017 Determination of residual solvents in docetaxel by headspace gas chromatography. Arab. J. Chem. 36, S2479–S2484. (10.1016/j.arabjc.2013.09.014)

[16] Ma T, Zhang YS, Chen AZ, Ju J, Gu C-W, Kankala RK, Wang S-B 2017 Carbon dioxide-assisted bioassembly of cell-loaded scaffolds from polymeric porous microspheres. J. Supercrit. Fluid. 120, 43–51. (10.1016/j.supflu.2016.10.010)

[17] Autian J 1975 Biological model systems for the testing of the toxicity of biomaterials. In Polymers in medicine and surgery, vol. 8 (eds Kronenthal RL, Oser Z, Martin E), pp. 181–203. New York, NY: Plenum Press.

[18] Ma J, Liu Z, Wang F, Liu Z, Wang F, Zhou Q, Feng C, Li F 2013 Preparation of a new radiolabeled biomaterial and its biodistribution in mice. J. Bionic. Eng. 10, 514–521. (10.1016/S1672-6529(13)60245-0)

[19] Taaca KL, Vasquez MR Jr 2018 Hemocompatibility and cytocompatibility of pristine and plasma-treated silver-zeolite-chitosan composites. Appl. Surf. Sci. 432, 324–331. (10.1016/j.apsusc.2017.04.034)

[20] Feldman AT, Wolfe D 2014 Tissue processing and hematoxylin and eosin staining. Methods Mol. Biol. 1180, 31–43. (10.1007/978-1-4939-1050-2_3) 25015141

[21] Cardiff RD, Miller CH, Munn RJ 2014 Manual hematoxylin and eosin stain of mouse tissue section. Cold Spring Harb. Protoc. 2014, 655–658. (10.1101/pdb.prot073411) 24890205

[22] Fischer AH, Jacobson KA, Rose J, Zeller RR 2008 Hematoxylin and eosin staining of tissue and cell sections. Cold Spring Harb. Protoc. 2008, pdb prot4986 (10.1101/pdb.prot4986) 21356829

[23] Kamiya Y, Yamaki T, Uchida M, Hatanaka T, Kimura M, Ogihara M, Morimoto Y, Natsume H 2017 Preparation and evaluation of PEGylated poly-L-ornithine complex as a novel absorption enhancer. Biol. Pharm. Bull. 40, 205–211. (10.1248/bpb.b16-00781) 28154261

[24] Takahashi H et al. 2017 An exploratory study on the anti-inflammatory effects of fucoidan in relation to quality of life in advanced cancer patients. Integr. Cancer Ther. 17, 282–291. (10.1177/1534735417692097) 28627320 PMC6041928

[25] Chen CH, Lin YS, Wu SJ, Mi F-L 2018 Mutlifunctional nanoparticles prepared from arginine-modified chitosan and thiolated fucoidan for oral delivery of hydrophobic and hydrophilic drugs. Carbohyd. Polym. 193, 163–172. (10.1016/j.carbpol.2018.03.080) 29773368

[26] Huang YC, Chen JK, Lam UI, Chen SY 2014 Preparing, characterizing, and evaluating chitosan/fucoidan nanoparticles as oral delivery carriers. J. Polym. Res. 21, 415 (10.1007/s10965-014-0415-6)

